# Cyclooxygenase-2 selective inhibitors increase the risk of alveolar osteitis: A systematic review and meta-analysis

**DOI:** 10.4317/medoral.27577

**Published:** 2026-01-24

**Authors:** Mario Alberto Isiordia-Espinoza, Adriana Hernández-Gómez, Ronell Bologna-Molina, Nicolás Serafín-Higuera, Nelly Molina-Frechero, Eduardo Gómez-Sánchez, Sandra López-Verdín, Juan Manuel Guzmán-Flores

**Affiliations:** 1 División de Ciencias Biomédicas, Centro Universitario de los Altos, Universidad de Guadalajara, Tepatitlán de Morelos 47620, Jalisco, México; 2 Facultad de Odontología, Universidad Juárez del Estado de Durango, Durango 34000, México; 3 Facultad de Odontología Mexicali, Universidad Autónoma de Baja California, Mexicali 21040, Baja California, Mexico; 4 Departamento de Salud, Laboratorio de Cariología y Medicina Oral, Universidad Autónoma Metropolitana-Xochimilco, Ciudad de México 04960, Mexico; 5 Centro Universitario de Ciencias de la Salud, Universidad de Guadalajara, Guadalajara 44340, Jalisco, Mexico

## Abstract

**Background:**

The purpose of this systematic review was to determine the risk of alveolar osteitis with the use of traditional non-steroidal anti-inflammatory analgesics (NSAIDs) and COX-2 selective inhibitors compared to placebo using data from published clinical trials.

**Material and Methods:**

Embase and Medline/PubMed databases were employed to search for clinical trials. Articles that met the inclusion criteria were assessed for risk of bias using the Cochrane tool and data on alveolar osteitis were specifically extracted. Statistical analysis was performed using Review Manager 5.3 software.

**Results:**

The risk assessment for alveolar osteitis concerning traditional NSAIDs included 19 clinical trials (n=2888) while for COX-2 inhibitors included 13 clinical trials (n=3820). Overall statistical analysis and subgroup evaluation by medication indicated that traditional NSAIDs did not increase the risk of alveolar osteitis. On the other hand, subgroup statistical analysis for selective COX-2 inhibitors suggested that rofecoxib elevated a patient's risk of developing alveolar osteitis (n=947, I2=0%, Z=2.52, OR=1.89, 95% CI=1.15 to 3.10, p=0.01) compared with placebo. Sensitivity analysis of the rofecoxib data exhibited high statistical robustness.

**Conclusions:**

Data indicates that rofecoxib increases the risk of alveolar osteitis in patients undergoing tooth extraction.

## Introduction

The healing of a tooth socket after a simple exodontia or dental surgery has been studied to detect any alterations that could lead to postoperative complications, such as a bacterial or fungal infection and alveolar osteitis (1 - 4). Alveolar osteitis-commonly known as post-extraction alveolitis, dry socket, as well as necrotic socket-is a painful clinical condition that occurs during the initial days after tooth extraction (5 - 9). Clinically, it is characterized by the total or partial absence of a blood clot within the tooth socket, bare bone, severe nerve symptoms, and halitosis (6 - 9).

Multiple studies have identified various risk factors for alveolar osteitis, many of which remain controversial (5 - 8). Common risk factors frequently reported in literature for this condition are as follows: Trauma from surgical procedures (5 , 6), being a female patient (5), tobacco smoking (7), inadequate use of irrigants (5 , 9), excessive manipulation of the socket (5), presence of alveolar osteitis in the mandible rather than the maxilla (6), and bacterial infections (5 , 6). Use of certain medications has also been reported to pose a risk of alveolar osteitis to patients. This includes local anesthetic with vasoconstrictor (5 , 9), oral contraceptives (5 , 6), and antipsychotics (6).

NSAIDs are medications frequently used to manage common postoperative complications after tooth extraction surgery such as simple exodontia and third molar removal (10 - 12). This group of drugs can be classified according to their mechanism of action: Traditional NSAIDs, which inhibit the constitutive (COX-1) and inducible (COX-2) cyclooxygenase enzymes (13); oxicams, which are partially selective for COX-2 (14 , 15); and selective inhibitors of the COX-2 enzyme (16 , 17). The COX-2 enzyme plays a fundamental role in the inflammation of tissue surrounding the surgical area after tooth extraction and has a key function in the healing of soft tissue and alveolar bone (18 - 23). Using data from published clinical trials (24), our study aims to compare the risk of alveolar osteitis associated with the use of traditional NSAIDs and COX-2 selective inhibitors to a placebo or an untreated control group in patients undergoing a simple exodontia or a third molar surgery.

## Material and Methods

This quantitative systematic review was conducted in accordance with international guidelines for this type of study (25) and the Cochrane Handbook for Systematic Reviews of Interventions Version 5.1.0 (2011) (26).

Database Search

This systematic review includes articles published between January 1990 and December 2024. Embase and Medline/PubMed databases were used to search for clinical trials reporting the efficacy of traditional NSAIDs and/or COX-2 inhibitors after tooth extraction using the following keywords: "Aspirin AND placebo AND third molar surgery", "diclofenac AND placebo AND third molar surgery", "ibuprofen AND placebo AND third molar surgery", "ketorolac AND placebo AND third molar surgery", "naproxen AND placebo AND third molar surgery", "celecoxib AND placebo AND third molar surgery", "etoricoxib AND placebo AND third molar surgery", "parecoxib AND placebo AND third molar surgery", "rofecoxib AND placebo AND third molar surgery", "valdecoxib AND placebo AND third molar surgery", "lumiracoxib AND placebo AND third molar surgery", "aspirin AND placebo AND tooth extraction", "diclofenac AND placebo AND tooth extraction", "ibuprofen AND placebo AND tooth extraction", "ketorolac AND placebo AND tooth extraction", "naproxen AND placebo AND tooth extraction", "celecoxib AND placebo AND tooth extraction", "etoricoxib AND placebo AND tooth extraction", "parecoxib AND placebo AND tooth extraction", "rofecoxib AND placebo AND tooth extraction", "valdecoxib AND placebo AND tooth extraction", and "lumiracoxib AND placebo AND tooth extraction". In the Embase database, the study type ("Human", "Clinical trial", and "Controlled Clinical Trial") and publication type ("Clinical Trial") filters were used. In Medline/PubMed, the article type ("Clinical Trial" and "Controlled Clinical Trial") and Species (Humans) filters were used. Only studies in English were considered (PROSPERO ID: CRD42024627337).

This study includes clinical trials of patients undergoing simple exodontia or third molar surgery who were treated with either a traditional NSAID or a COX-2 inhibitor drug, compared to a placebo group or an untreated control group and who presented symptoms of alveolar osteitis. Clinical trials reporting patient loss greater than 20% were excluded.

Assessment of bias

The risk of bias in the included studies was assessed using the Cochrane tool for interventions (27 - 31). This tool allowed for the assessment of risk of bias in 5 key elements: "Selection, performance, attrition, reporting, and other" (sample size calculation) (27 - 31). Each clinical trial was assessed using this tool solely to determine the risk of bias, and no studies were excluded due to the high risk of bias. The evaluation was conducted by two professors with extensive experience in using this tool, which facilitated consensus between them on all evaluations (27 - 31).

Data extraction

Only data indicating the frequency of alveolar osteitis, alveolitis, or dry socket when treated using a traditional NSAID or a COX-2 inhibitor compared with a placebo/untreated group were extracted. Data was entered directly into the Review Manager 5.3 software for Windows. These meta-analyses were then printed and provided to two reviewers to confirm that the extracted information was correct.

Statistical analysis

Dichotomous data on alveolar osteitis were analyzed using the fixed effects method, and the Mantel-Haenszel weighting method, the Odds Ratio (OR) effect measure, and 95% confidence intervals (95%CIs) were used in all meta-analyses (25 - 27 , 32 , 33). Additionally, the absolute risk increase (ARI) and 95%CIs were calculated (34 - 36). A p-value&lt;0.05 was considered statistically significant in the overall tests or in the drug subgroup evaluation (OR&gt;1 within the 95% CIs) (25 - 27 , 32 , 33). A sensitivity analysis was performed for rofecoxib to observe any significant changes in the p-value (37 , 38). Funnel plots were performed to explore potential publication bias in clinical trials (26).

## Results

Database Search

A total of 711 articles were identified in the reviewed databases. After discarding duplicates, studies not related to the topic of interest, and studies in languages other than English, 326 scientific article abstracts were reviewed. Only 97 clinical trials were fully evaluated, of which only 25 were included in this systematic review (Figure S1: http://www.medicina.oral.com/carpeta/suppl1_27577).

Risk of bias assessment and qualitative evaluation

The risk of bias assessment showed that only three clinical trials had a low risk of bias (48 , 57 , 61), while the remainder of the included studies had a moderate risk of bias (Figure S2 and Figure S3: http://www.medicina.oral.com/carpeta/suppl1_27577) (39 - 47 , 49 - 56 , 58 - 60 , 62 , 63).

The parallel clinical trial was the main design of the included studies (24/25) (39 - 59 , 61 - 63). Most studies used a single dose (24/25) (39 - 44 , 46 - 63), and the oral administration route was the most used (21/25) (39 , 40 , 42 , 44 - 54 , 57 - 63). Table S1 (http://www.medicina.oral.com/carpeta/suppl2_27577) contains further details of the included studies.

Quantitative assessment

**Figure 1:**
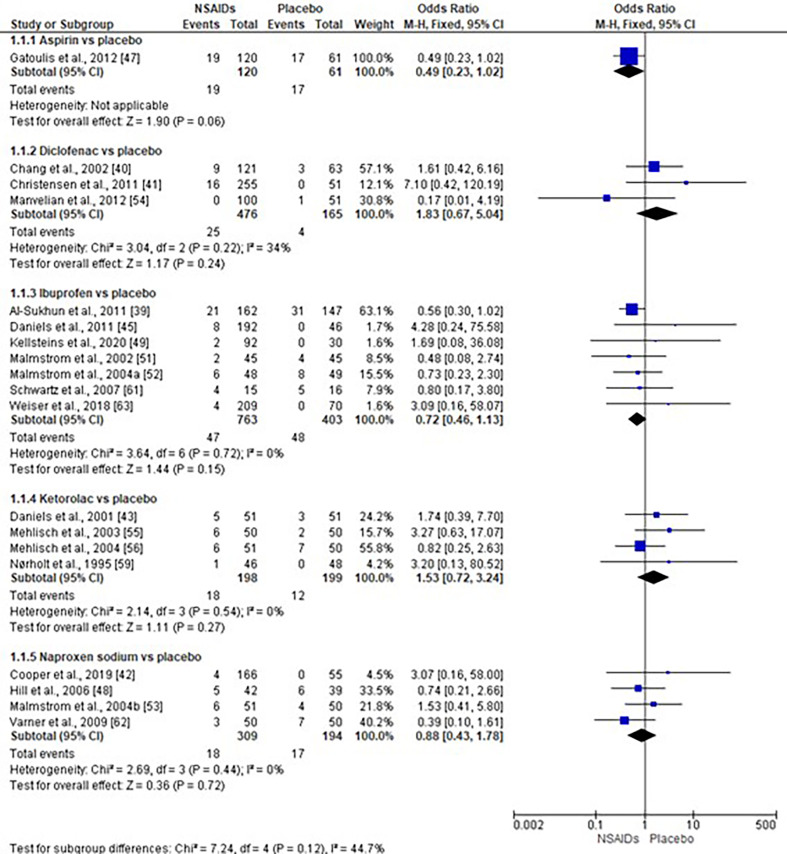
Risk of alveolar osteitis with the use of traditional NSAIDs.

The risk of alveolar osteitis associated with traditional NSAIDs compared with placebo was assessed using data from 19 clinical trials (n=2888) (39 - 43 , 45 , 47 - 49 , 51 - 56 , 59 , 61 - 63). The subgroup and the overall analyses showed no statistical difference in the risk of alveolar osteitis (Figure 1) (39 - 43 , 45 , 47 - 49 , 51 - 56 , 59 , 61 - 63).

1

**Figure 2:**
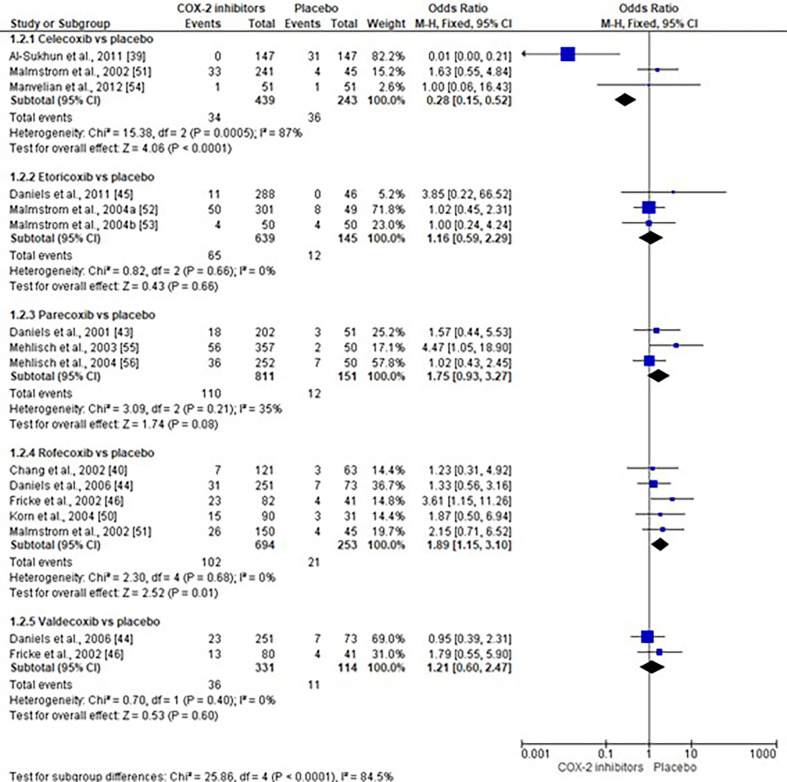
Risk of alveolar osteitis with the use of COX-2 inhibitors.

The risk of alveolar osteitis with selective COX-2 inhibitors compared with placebo was assessed using 13 clinical trials (n=3820) (39 , 40 , 43 - 46 , 50 - 56). Subgroup analysis showed that rofecoxib increases the risk of alveolar osteitis (n=947, I2=0%, Z=2.52, OR=1.89, 95% CI=1.15 to 3.10, p=0.01, Figure 2) (40 , 44 , 46 , 50 , 51), whereas overall results showed no statistical difference (39 , 40 , 43 - 46 , 50 - 56). Additionally, the ARI indicates that 6.40% (2.10% to 10.70%) of patients will experience adverse events with rofecoxib that would not have occurred with a placebo (40 , 44 , 46 , 50 , 51).

2

Sensitivity analysis

**Figure 3:**
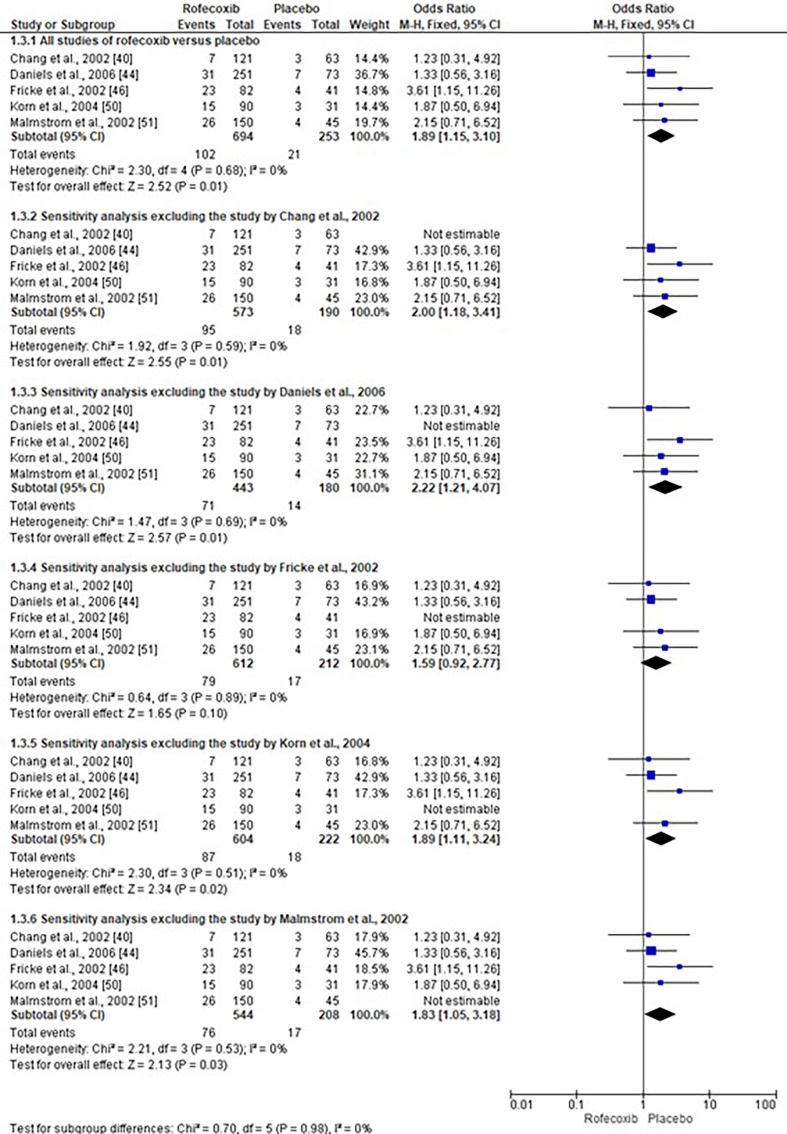
Sensitivity analysis of rofecoxib data.

The sensitivity analysis revealed no changes in p-values in both the overall and subgroup statistical analyses for the comparisons of traditional NSAIDs versus placebo and COX-2 inhibitors versus placebo (39 - 43 , 45 , 47 - 49 , 51 - 56 , 59 , 61 - 63). Figure 3 details the sensitivity analysis conducted on the data of rofecoxib versus placebo, demonstrating that the p-value remained consistently significant (40 , 44 , 46 , 50 , 51).

3

Publication bias

**Figure 4:**
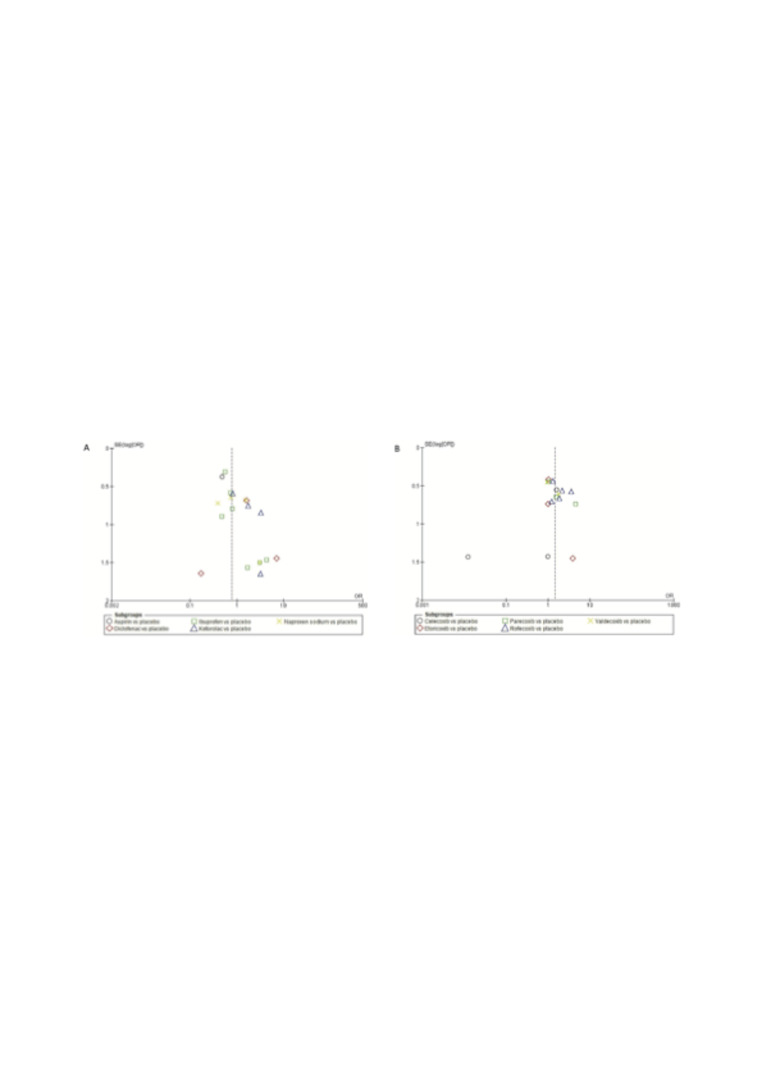
Publication bias.

Visual evaluation of the funnel plots revealed asymmetry for traditional NSAIDs (ketorolac) (Figure 4A) as well as for COX-2 inhibitors (celecoxib) (Figure 4B).

4

## Discussion

According to the biomedical literature reviewed by the authors, this is the first systematic review and meta-analysis conducted with the aim of evaluating the risk of alveolar osteitis following the administration of traditional NSAIDs and selective COX-2 inhibitors after single tooth extraction or third molar surgery. The results indicate an increased risk of alveolar osteitis in patients receiving rofecoxib compared with those receiving a placebo. Previously, the clinical trial by Al-Sukhun et al., 2011(39) was conducted with the specific purpose of evaluating the risk of alveolar osteitis from the use of a selective COX-2 inhibitor (celecoxib), ibuprofen, and placebo after mandibular tooth extraction. The results of that study showed that the use of these medications did not increase the risk of alveolar osteitis in the study population (39). Other studies included in this systematic review did not directly evaluate the risk of alveolar osteitis from the administration of traditional NSAIDs and/or selective COX-2 inhibitors, but alveolar osteitis was documented as an adverse effect after pharmacological treatment of inflammation, postoperative pain, and trismus after these surgical procedures (40 - 63).

Some traditional NSAIDs, such as diclofenac (40 , 41 , 54) and ketorolac (43 , 55 , 56 , 59), and most COX-2 inhibitors showed an OR greater than 1 with a 95%CI less than one and a p-value showing no statistical difference compared with the placebo (39 - 43 , 45 , 47 - 49 , 51 - 56 , 59 , 61 - 63). However, these observations suggest that increasing the number of studies and sample size of this quantitative systematic review could reveal an increased risk of alveolar osteitis with the use of these medications in patients undergoing the aforementioned surgical procedures. Notably, the association between rofecoxib use and an increased risk of alveolar osteitis, compared to placebo, demonstrated high statistical robustness as indicated by sensitivity analysis (Figure 4) (40 , 44 , 46 , 50 , 51).

Scientific evidence suggests that the use of selective COX-2 inhibitors and traditional NSAIDs may have significant effects on the healing process of both soft and hard tissues. Preclinical studies have demonstrated that COX-2 inhibition can alter inflammatory responses, impair vascularization, and delay wound healing, which raise concerns regarding its potential impact on postoperative recovery in oral surgical procedures. A study by Ren et al., 2013 (64), evaluated the effect of parecoxib, a selective COX-2 inhibitor, on skin flaps in animal models. Their results showed that the group receiving parecoxib exhibited extensive areas of tissue necrosis compared with the placebo group. This effect was attributed to COX-2 inhibition leading to a downregulation of Vascular Endothelial Growth Factor (VEGF), reducing angiogenesis and impairing oxygen supply, which consequently increased oxidative stress and tissue necrosis (64). Conversely, in the placebo group, normal VEGF expression contributed to adequate angiogenesis, endothelial nitric oxide synthase activation, leukocyte migration, and apoptosis regulation, ultimately supporting better tissue oxygenation and reduced necrosis (64 , 65). Furthermore, Guo et al., 2023 demonstrated that celecoxib affects bone metabolism and angiogenesis (-catenin decreased and VEGF) in patients with axial spondyloarthritis (66). Matsuyama et al., 2018, using the mouse pre-osteoblast cell line MC3T3-E1, demonstrated that celecoxib and valdecoxib inhibit osteoblast differentiation through phosphorus-dependent intracellular signaling, thus modifying bone remodeling (67). Additionally, Daluiski et al., 2006 that celecoxib decreases the osteogenic potential of Saos-2 cells (Saos-2 osteoprogenitor cell lines) (68). Futagami et al., 2002 (69) demonstrated that both a selective COX-2 inhibitor (NS-398) and a traditional NSAID (indomethacin) delayed wound healing in rat skin. Notably, the delay induced by NS-398 occurred during the early stages of re-epithelialization, coinciding with increased COX-2 expression, suggesting a critical role for this enzyme in the initial wound healing phase (69). Furthermore, a systematic review by Solaiman et al., 2024 (70) assessing wound healing in animal models undergoing knee surgery reported consistent delays in both soft and hard tissue repair associated with NSAID administration. In addition to its effects on soft tissue healing, COX-2 inhibition may also interfere with bone regeneration. A study by Lu et al., 2017 (71) showed that prostaglandin E2 (PGE2), a downstream product of COX-2, promotes early osteogenesis by stimulating mesenchymal stem cells. However, when celecoxib, a selective COX-2 inhibitor, was introduced, PGE2 expression was downregulated, leading to decreased bone mineralization in the tested samples (71). The researchers concluded that this mechanism could be a significant adverse effect of NSAIDs and COX-2 inhibitors on fracture healing and bone regeneration (71).

Considering these findings, it is hypothesized that the mechanisms of action of traditional NSAIDs and selective COX-2 inhibitors may lead to similar alterations in periodontal soft tissues and the alveolar bone, potentially increasing the risk of alveolar osteitis in patients receiving these drugs following tooth extraction or oral surgery. Inhibition of the COX-2 enzyme suppresses postoperative inflammation, which is a key process for wound healing. This may affect the normal process of new blood vessel formation, delay epithelialization of the dental alveolus, and reduce osteogenesis, which could potentially contribute to a higher incidence of alveolar osteitis. Further clinical and experimental research is necessary to elucidate the precise effects of these pharmacological agents on the healing dynamics of the dental alveolus and to establish evidence for their use in dental and oral surgical settings. These drugs are added to other risk factors previously identified in the literature. In other words, we do not assume they are the sole cause of alveolar osteitis; rather, in conjunction with other known or unknown risk factors, they could significantly contribute to the development of this clinical condition.

The key strength of this systematic review include its rigorous methodology-encompassing the search strategy and statistical analysis-the low to moderate risk of bias in the included studies, and the proposed explanation for the potential biological mechanism that increases the risk of alveolar osteitis, which is supported by various preclinical studies. Nevertheless, two important limitations are the low number of clinical trials included in each specific meta-analysis, and the classification of alveolar osteitis as an adverse event rather than an adverse reaction in the included studies. A clinical trial is regarded as the most important type of study demonstrating a cause-effect relationship. In such trials, all groups must be equal in all aspects except for the intervention received; in this specific case, the intervention is pharmacological treatment (72 , 73). Consequently, alveolar osteitis should have been directly related to it. Furthermore, we emphasize that the funnel plots in our systematic review were based on fewer than 10 articles per drug, contrary to recommendations for their use in assessing publication bias (26). Another limitation is that it was restricted to articles published in English.

## Conclusions

The data from this systematic review and meta-analysis indicate that rofecoxib, a selective COX-2 inhibitor, may increase the risk of alveolar osteitis in patients undergoing simple exodontia or third molar surgery. In this context, it is notable that most selective COX-2 inhibitors exhibited a trend toward an increased risk of alveolar osteitis, although no statistical significance was observed. For this reason, future high-quality primary studies are needed to strengthen the evidence on this association.

## Data Availability

Declared none.
